# *Rickettsia felis*, West Indies

**DOI:** 10.3201/eid1603.091431

**Published:** 2010-03

**Authors:** Patrick J. Kelly, Helene Lucas, Marina E. Eremeeva, Kathryn G. Dirks, Jean Marc Rolain, Charles Yowell, Reginald Thomas, Trevrone Douglas, Gregory A. Dasch, Didier Raoult

**Affiliations:** Ross University School of Veterinary Medicine, St. Kitts, West Indies (P.J. Kelly, H. Lucas); Centers for Disease Control and Prevention, Atlanta, Georgia, USA (M.E. Eremeeva, K.G. Dirks, G.A. Dasch); Unité de Recherche sur les Maladies Infectieuses et Tropicales Emergentes, Marseille, France (J.M. Rolain, D. Raoult); University of Florida, Gainesville, Florida, USA (C. Yowell); Division of Agriculture, Roseau, Dominica, West Indies (R. Thomas, T. Douglas)

**Keywords:** Rickettsia felis, West Indies, St. Kitts, Dominica, rickettsia, letter

**To the Editor:** A spay–neuter (sterilization) program for feral cats from Basseterre, the capital of the Caribbean Island St. Kitts, found that most (45/58; 66%) cats had antibodies to spotted fever group rickettsiae (SFGR). The antibodies were detected with *Rickettsia rickettsii* antigen in a standard microimmunofluorescence assay (*1*). Titers for 13 (20%) cats were >320.

Most SFGR are transmitted by ticks, but because of their grooming habits, cats seldom have many ticks (*2*), and we did not find any ticks on the cats we saw through the program. We did, however, commonly find cat fleas, *Ctenocephalides felis*, which are the main vector of *R. felis*, a recently described member of the SFGR. *R. felis* seems to be apathogenic in cats (*3*) but is the agent of flea-borne spotted fever in humans (*4*). Although *R. felis* has been reported from North and South America, Europe, Africa, the Middle-East, and Oceania (*4*), its presence in the Caribbean islands has not been established. To provide this information we tested DNA extracted with the QIAamp DNA Mini-Kit (QIAGEN, Valencia, CA, USA) from *C. felis* fleas preserved in 70% ethanol.

Of 57 (19%) *C. felis* fleas from St. Kitts, 11 were positive for *R. felis* DNA when tested by PCR using primers targeting SFGR *omp*A (*5*) or TaqMan assay using primers targeting *glt*A and a probe specific for the organism (*6*,*7*). For a negative control we used distilled water; for a positive control we used DNA from *R. montanensis* cultures or recombinant control plasmids constructed by amplifying target fragments from *R. typhi* strain Wilmington and *R. felis* strain LSU (*7*). The sequences of the *omp*A and *glt*A amplicons obtained had 100% nucleotide sequence similarity with homologous fragments of the type reference isolate *R. felis* URRxCal2. We used the National Center for Biotechnology Information basic local alignment sequence tool, BLAST (www.ncbi.nlm.nih.gov/blast/Blast.cgi).

To determine whether *R. felis* occurs on another Caribbean island, we tested 32 *C. felis* fleas from Dominica and found 1 (3%) to be positive by PCR when primers targeting *omp*A were used. The sequence obtained was also identical to that of *R. felis* URRxCal2.

Our study provides further evidence that cats can be sentinels for the presence of rickettsiae (*1*). However, although rickettsemia can develop in cats experimentally infected with *R. felis* (*3*), no compelling evidence shows that cats help maintain the organism or transmit it to humans (*8*,*9*). Rather, it appears that *C. felis* fleas, which are also commonly found on dogs and to a lesser extent other mammals, are the major reservoir hosts and vectors of infection, although the exact mechanisms are unknown (*10*). Our study also expands the known distribution of *R. felis* and should alert healthcare workers who see residents of or vacationers from the Caribbean islands of the possibility of flea-borne spotted fever in their patients.
